# NAT10 is upregulated in hepatocellular carcinoma and enhances mutant p53 activity

**DOI:** 10.1186/s12885-017-3570-4

**Published:** 2017-08-31

**Authors:** Qijiong Li, Xiaofeng Liu, Kemin Jin, Min Lu, Chunfeng Zhang, Xiaojuan Du, Baocai Xing

**Affiliations:** 1 Department of Hepatobiliary Oncology, Sun Yat-Sen University Cancer Center, 651 Dongfeng Road East, Guangzhou, Guangdong 510060 China; 2 Department of Cell Biology, School of Basic Medical Sciences, Peking University Health Science Center, Beijing, 100191 China; 3 Hepatopancreatobiliary Surgery Department I, Key Laboratory of Carcinogenesis and Translational Research, Ministry of Education, Peking University School of Oncology, Beijing Cancer Hospital and Institute, 52 Fucheng Road, Haidian District, Beijing 100142 China; 4 Department of Pathology, School of Basic Medical Sciences, Peking University Health Science Center, Beijing, 100191 China

**Keywords:** NAT10, Hepatocellular carcinoma, Prognosis, Mutant p53, Stability

## Abstract

**Background:**

N-acetyltransferase 10 (NAT10) is a histone acetyltransferase which is involved in a wide range of cellular processes. Recent evidences indicate that NAT10 is involved in the development of human cancers. Previous study showed that NAT10 acetylates the tumor suppressor p53 and regulates p53 activation. As *Tp53* gene is frequently mutated in hepatocellular carcinoma (HCC) and associates with the occurrence and development of HCC, the relationship between NAT10 and HCC was investigated in this study.

**Methods:**

Immunohistochemistry (IHC) and western blot analysis were performed to evaluate the NAT10 expression in HCC. Immunoprecipitation experiments were performed to verify the interaction of NAT10 with mutant p53 and Mdm2. RNA interference and Western blot were applied to determine the effect of NAT10 on mutant p53. Cell growth curve was used to examine the effect of NAT10 on HCC cell proliferation.

**Results:**

NAT10 was upregulated in HCC and increased NAT10 expression was correlated with poor overall survival of the patients. NAT10 protein levels were significantly correlated with p53 levels in human HCC tissues. Furthermore, NAT10 increased mutant p53 levels by counteracting Mdm2 action in HCC cells and promoted proliferation in cells carrying p53 mutation.

**Conclusion:**

Increased NAT10 expression levels are associated with shortened patient survival and correlated with mutant p53 levels. NAT10 upregulates mutant p53 level and might enhance its tumorigenic activity. Hence, we propose that NAT10 is a potential prognostic and therapeutic candidate for p53-mutated HCC.

## Background

Hepatocellular carcinoma (HCC) is one of the most prevalent malignancies throughout the world and has been the third leading-cause of cancer-related death worldwide [1]. The mechanisms involved in the development and progression of HCC remain poorly understood. In recent years, the relationship between the somatic mutations and HCC was further elucidated by the identification of crucial genes and pathways in HCC [2]. Wnt/β-catenin was found to be the most frequently mutated pathway, while the p53 pathway was considered to be the second most frequently mutated pathway in HCC, with the occurrence about 21% in HCC [3–5]. Nevertheless, the mechanism of how genetic changes lead to pathological and physiological changes is still unclear. Therefore, the further exploration of the mechanisms of how genetic mutations lead to hepatic tumorigenesis require further study.

p53, a key tumor repressor, plays a vital role in various critical cellular processes, including DNA repair, cell cycle regulation, apoptosis induction, etc. [6, 7]. *TP53* mutations were frequently observed in human cancers and various studies have indicated that some mutant p53 proteins facilitate tumorigenesis [8, 9]. Mutant p53 exhibits a wide range of distinct change of genetic structures which lead to altered heat stability, and lose the ability to bind p53-responding elements and transactivate downstream genes [10]. Moreover, mutant p53 exhibits characteristic of oncogene such as loss of cell growth control and gain functions in promoting tumorigenesis [11–13]. Previous studies found that the *Tp53* gene mutations were frequently observed in HCC, and these mutations were correlated with stage and prognosis of tumor [4, 5]. Recent study has shown that compared with HCC patients without detectable p53 mutations, patients carrying *Tp53* mutations suffer poor prognosis of higher recurrence rate and shorter overall survival [14]. Given the pivotal role of mutant p53 in tumorigenesis, some strategies to target p53 mutations have been developed in order to treat HCC [15–19]. Accordingly, these findings indicate that mutant p53 is a relatively key role in the pathogenesis of HCC. Therefore, further study to understand the modulations and functional changes of mutant p53 in HCC is essential.

N-acetyltransferase 10 (NAT10; named as hALP as well), is a member of the Gcn5-related N-acetyltransferase family of histone acetyltransferases (HATs). Previous study showed that truncated recombinant NAT10 (amino acids 164–834) acetylates calf thymus histones in vitro [20]. NAT10 is located in the nucleolus and involved in the regulation of telomerase activity, rRNA transcription, and cytokinesis [21–24]. The NAT10 inhibitor, remodelin, can be used to ameliorate laminopathies through correcting nuclear architecture and attenuating senescence [25]. Recent reports demonstrated that NAT10 enhances p53 activity through acetylating p53 and counteracting Mdm2 action in response to DNA damage [26]. Given its pivotal role in p53 activation, the aim of this study was to investigate whether NAT10 can regulate mutant p53 activity**.**

## Methods

### Cell culture and transfections

The hepatoma cell lines Huh7 (mutant p53 Y220C) was obtained from COBIOER BIOSCIENCES CO., LTD (COBIOER, Nanjing, China) and HepG2 (wild-type p53), MHCC-97H (R249S), MHCC-97 L (R249S) and the normal hepatic cell line LO2 (wild-type p53) were gifts from Prof. Curtis C. Harris. Liver cancer cells were cultured and maintained in Dulbecco’s modified Eagle’s medium supplemented with 10% fetal bovine serum at 37 °C in a humidified atmosphere containing 5% CO_2_. Cells were transfected with plasmid DNA or siRNA duplexes by using Lipofectamine® 2000 (Invitrogen, CA, USA) according to the manufacturer’s protocol. For silencing NAT10 expression, a small interfering RNA (siRNA) targeting NAT10 (sequence: 5′-CAGCACCACUGCUGAG AAUAAGA-3′), Mdm2 (sequence: 5′-AAGCCAUUGCUUUUGAAGUUA-3′) was synthesized, together with the control siRNA (5′-ACUACCGUUGUUAUAGGUG-3′; Shanghai GenePharma Co., Ltd).

### Plasmids and antibodies

FLAG-tagged NAT10 and NAT10 mutants were cloned into pCI-neo. Anti-p53 (DO-1), anti-actin (C-11), anti-Mdm2 (SMP14) and anti-p21 (817) antibodies were purchased from Santa Cruz Biotechnology, Inc. Anti-Flag (F3165) antibody was purchased from Sigma. Anti-NAT10 antibody was a gift from Dr. B. Zhang.

### Preparation of cellular extracts

Preparation of cellular extracts was performed as described previously [26]. In Brief, cells were harvested and washed with PBS. Then, cells were lysed in ice-cold H lysis buffer A (10 mM Tris-HCl pH 7.4, 10 mM KCl, 2 mM MgCl_2_, 0.05% Triton™ X-100, 1 mM DTT, 1 mM EDTA and fresh proteinase inhibitors). Next, the nuclear pellet was collected after centrifugation for 10 min at 2000 rpm, and the supernatant was collected as the cytoplasmic extract. The crude nuclear pellet was suspended in T Lysis Buffer (20 mM HEPES pH 7.9, 420 mM NaCl, 0.2 mM EDTA, 1.5 mM MgCl_2_, 0.5 mM DTT, and 10% glycerol with protease inhibitor mixture) and swollen at 4 °C for 30 min. The homogenate was centrifuged for 15 min at 12000 rpm. Nuclear and cytoplasmic fractions were subjected to Western blot using the indicated antibodies.

### Immunoblotting

Total proteins were extracted and immunoblotting was performed as the standard procedures. Then, the immunoreactivity was detected with ECL Western blot Detection Reagent (GE Healthcare).

### Immunoprecipitation assay

Immunoprecipitation assay was performed as described previously [27]. In brief, Huh7 cellular lysates were prepared in lysis buffer A (25 mM Tris-HCl pH 7.5, 100 mM KCl, 1 mM DTT, 2 mM EDTA, 0.5 mM phenylmethylsulfonyl fluoride, and 0.1% Nonidet P-40). Cellular extracts were obtained by centrifugation for 30 min at 12000 rpm. Specific antibodies were incubated with 15 ul protein A and G beads (Amersham Biosciences) in IPP500 (500 mM NaCl, 10 mM Tris-HCl pH 8.0, 0.1% Nonidet P-40). Coupled beads were incubated with cellular extracts for 2 h at 4 °C. After extensive washes, the precipitated proteins were subjected to Western blotting.

### Cell growth assay

Cell growth curve was analyzed using the Cell Counting Kit-8 (CCK-8, Dojindo) according to the manufacturer’s directions. Briefly, Huh7 or MHCC-97 L cells were transfected with the indicated siRNAs (500 cells per well) and grew in 10% serum containing media. Cell numbers were estimated at day 0, 1, 2, 3, 4 and 5. The growth curve shows the mean ± standard deviation from three technical replicates.

### Patients and tumor tissues

Human HCC tissues and adjacent noncancerous tissues for western blotting were obtained from 19 patients with HCC who underwent tumor resection at the Beijing Cancer Hospital. After resection, specimens were rinsed thoroughly in ice-cold normal saline and stored in liquid nitrogen.

Sections were obtained from 119 formalin-fixed, paraffin-embedded human HCC tissues and corresponding non-cancerous tissues of the same patient undergoing surgical resection without prior neoadjuvant therapy between January 2003 and October 2006 in the Beijing Cancer Hospital. The clinico-pathological patient characteristics are summarized in Table 1.

**Table 1:** Correlations between NAT10 expression in HCCs and the clinicopathologic factors

	NAT10 expression level (Score)						
		0	1	2	3	Total	*p*
Age (years)	<=60	9	6	15	59	89	0.598
	>60	2	1	8	19	30	
Tumor Size (cm)	<5	10	6	13	33	62	0.005
	> = 5	1	1	10	45	57	
Serum AFP (ng/ml)	<=20	4	5	10	27	46	0.266
	>20	7	2	13	51	73	
Tumor Number	=1	10	7	20	60	97	0.287
	>1	1	0	3	18	22	
Lymph node metastasis	No	11	7	21	75	114	0.579
	Yes	0	0	2	3	5	
Tumor encapsulation	No	8	2	13	35	58	0.195
	Yes	3	5	10	43	61	
Vascular invasion	No	11	7	18	52	88	0.033
	Yes	0	0	5	26	31	
Edmondson-Steiner grade	ES = 1 ~ 2	8	6	19	55	88	0.597
	ES = 3 ~ 4	3	1	4	23	31	

NAT10 expression was determined in 119 HCCs samples by immunohistochemistry as described in the Methods. The correlations between the expression levels of NAT10 and clinico-pathological factors of HCCs were evaluated by the Mann-Whitney U test. We concluded that NAT10 expression was correlated with tumor size and vascular invasion (*p* < 0.05). However, NAT10 expression was not correlated with other factors such as age, α-fetoprotein (AFP) levels, capsular formation, tumor number, margin status, and Edmondson-Steiner grade

### Immunohistochemistry assay

Sections (4 μm thick) were dewaxed in xylene and gradually rehydrated gradually. After antigen retrieval, endogenous peroxidases were blocked with 3% hydrogen peroxide. Then, the sections were incubated with 10% goat serum for 30 min at room temperature. Sections were incubated with rabbit anti-NAT10 polyclonal antibody at 4 °C overnight and then with HRP-conjugated goat anti-rabbit IgG (Zhongshan Golden bridge Biotechnology, Beijing, China) at 37 °C for 30 min. Immunocomplexes were visualized by using 3,3-diaminobezidine (DAKO, CA, USA). Slides were counterstained with light hematoxylin, dehydrated, and cover-slipped.

Histological slides were assessed by two independent observers, including an experienced pathologist, blinded to all clinical, pathological, and outcome information. The score discrepancies were discussed to achieve a consensus. Immunostaining was categorized into four groups: negative (0 score), 0%–10% positive cells; faintly positive (1 score), 10%–25% positive cells; moderately positive (2 scores), 25–50% positive cells; and highly positive (3 scores), ≥50% positive cells.

### Statistical analysis

All statistical analyses were carried out using SPSS version 17 (SPSS Inc., Chicago, IL, USA) and GraphPad Prism (GraphPad Software). All data are shown as mean ± standard deviation. To compare the experimental groups, Student’s *t*-test and one-way analysis of variance were used. Associations between NAT10 immunohistochemical staining and clinico-pathological variables were analyzed using the Mann-Whitney U test. Survival was estimated using the Kaplan–Meier method, and the difference between the survival curves was analyzed using the log-rank test. Univariate and multivariate survival analyses were performed using the Cox proportional hazards model.

## Results

### NAT10 is upregulated in HCC patients and is correlated with shorter survival

**Fig. 1:**
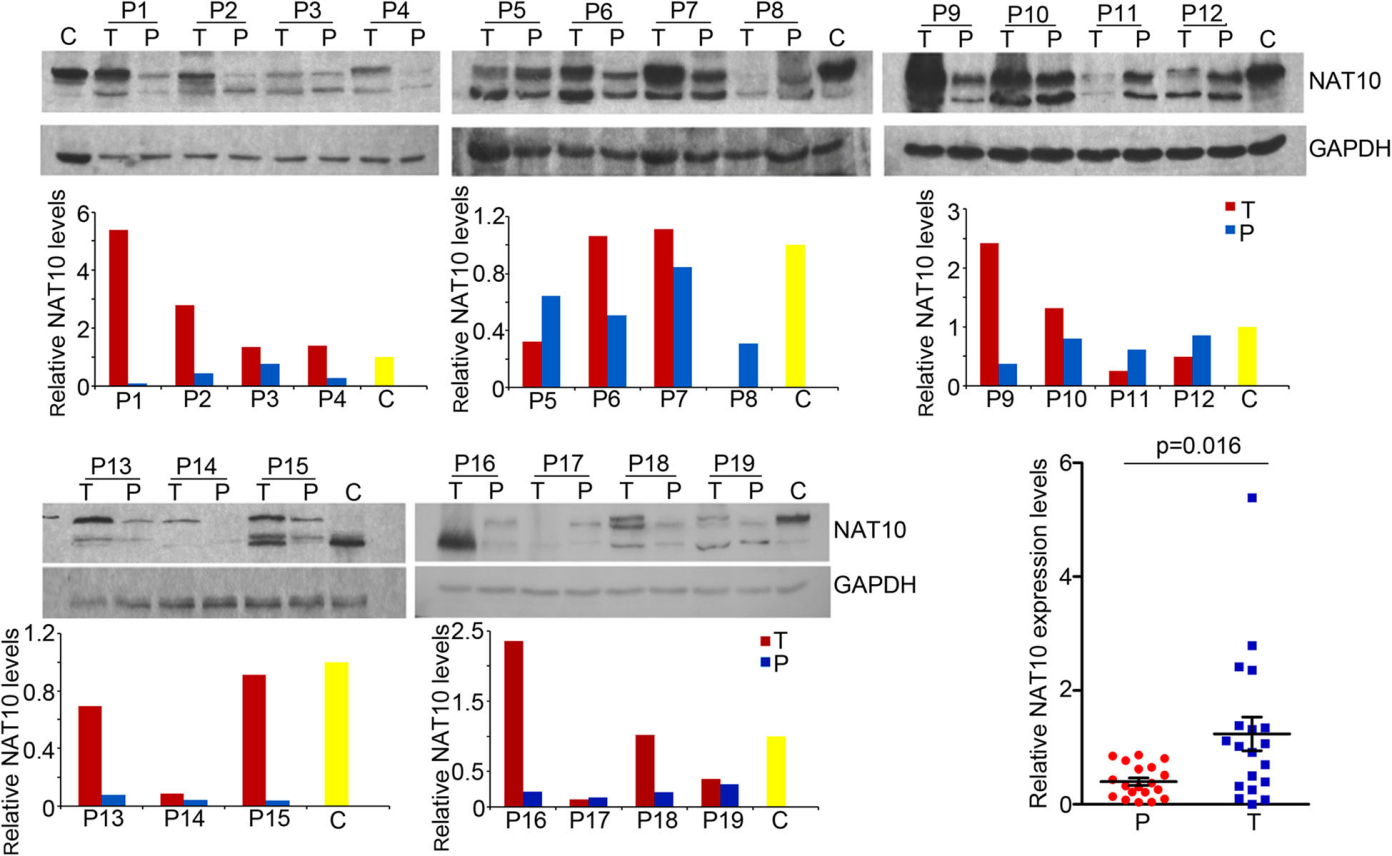
N-acetyltransferase (NAT10) is upregulated in human hepatocellular carcinoma (HCC). Immunoblotting revealed higher NAT10 protein in 14 of 19 tumor samples than in the respective matched pericancerous tissues (T, tumor; P, pericancerous tissue). Glyceraldehyde-3-phosphate dehydrogenase (GAPDH) was used as a loading control

To determine the significance of NAT10 in hepatocellular carcinomas, we performed immunoblotting on human HCC tissues and their matched noncancerous tissues. Fourteen of 19 (73.7%) tumor samples showed increased NAT10 protein levels compared with their respective paired noncancerous tissue (Fig. 1a). These data indicated a positive correlation of NAT10 expression with HCC.

**Fig. 2:**
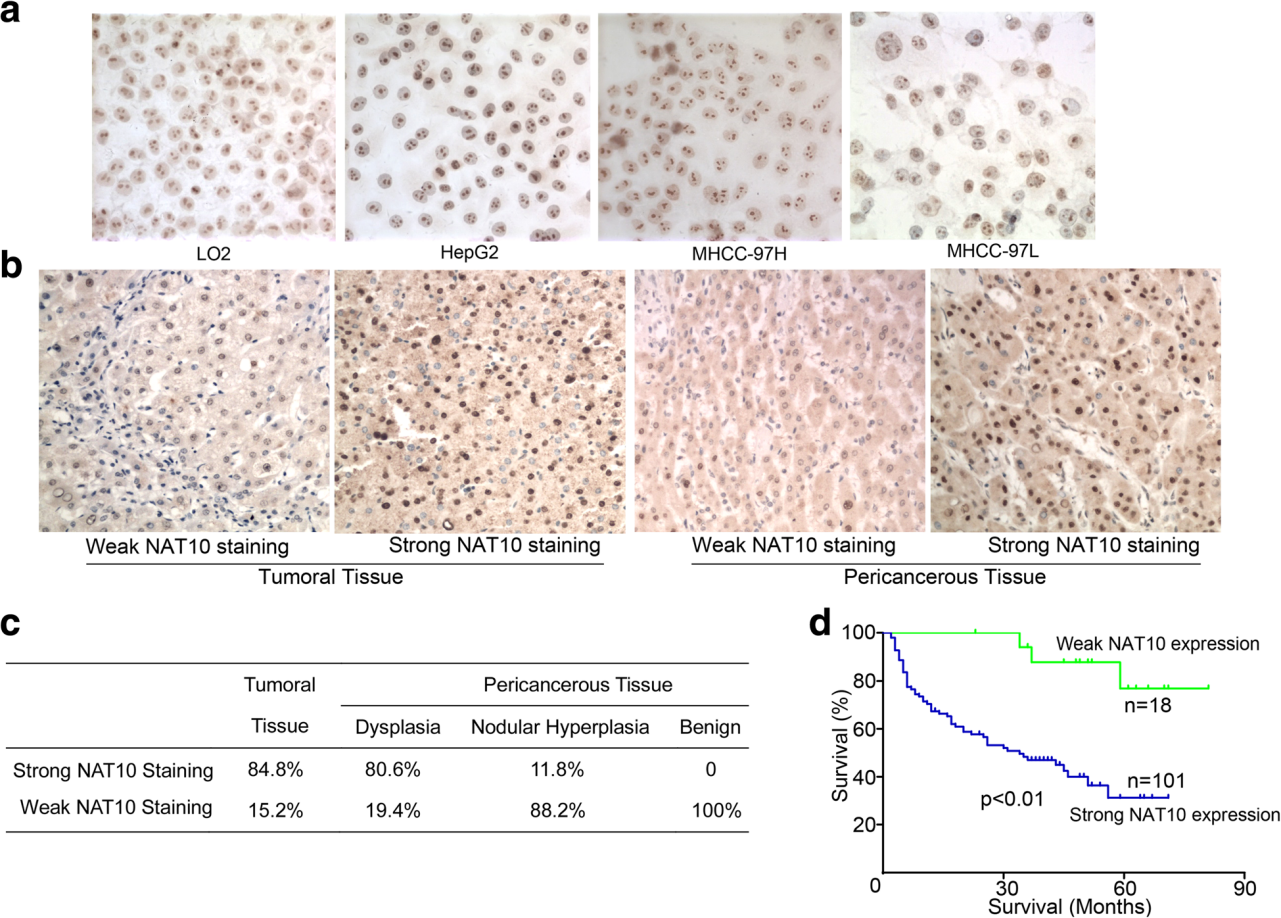
Increased NAT10 expression levels are associated with shortened survival of HCC patients. **a** Representative immunohistochemical staining of NAT10 in human HCC cells (magnification, ×400). **b** Representative immunohistochemical staining of NAT10 in adjacent noncancerous tissues and HCC tissues (magnification, ×200). **c** Summary of NAT10 expression in human HCC tissues and noncancerous tissues. **d** Overall survival of HCC patients with different levels of NAT10 expression by Kaplan-Meier analysis

Next, we investigated the correlation of NAT10 expression with HCC progression. Immunohistochemical staining was performed to evaluate NAT10 expression on primary human tumors from a large cohort of HCC patients (*n* = 119). Among these 119 patients, all biopsy specimens contained both tumors and matched non-tumorous tissues. Consistent with our previous study, NAT10 was expressed in the nuclei of human HCC tumor cells (Fig. 2a). For further evaluation of the expression level of NAT10, the staining level was graded and scored from 0 to 3. According to the staining score, all patients was subgrouped as weak expression (staining score 0–1) versus strong expression (staining score 2–3). Strong expression of NAT10 was detected in 101 of 119 cases (84.8%) of HCC tumor tissues, whereas NAT10 expression was not detected in their benign counterparts (Fig. 2b and c). Thus, NAT10 expression was significantly upregulated in HCC tumor tissues compared with their non-tumorous counterparts.

The correlation between NAT10 expression and clinico-pathological variables was analyzed using SPSS version 17. As shown in Table 1, NAT10 expression was significantly correlated with vascular invasion (*p* < 0.05). However, NAT10 expression did not correlate significantly with age, α-fetoprotein (AFP) levels, capsular formation, tumor number, margin status and Edmondson-Steiner grade. In addition, as indicated by Kaplan-Meier analysis, high level expression of NAT10 was associated with shorter overall survival (OS; Fig. 2d) in our cohort (*p* < 0.01). According to this result, we further investigated whether NAT10 expression can affect the prognosis of HCC patients independently. Univariate analysis by Cox-regression revealed that 5 prognostic factors affecting OS: NAT10 expression level, tumor size, tumor number, microvascular invasion and lymph node metastasis. Multivariate analysis by cox-regression revealed that NAT10 expression, tumor number, microvascular invasion, and lymph node metastasis were independent prognostic factors of OS (Table 2). These data demonstrated that NAT10 is an independent prognostic factor for HCC patients.

**Table 2:** Univariate and multivariate analyses of factors associated with prognosis in 119 HCCs

Clinicopathological characteristics		N	Univariable analysis		Multivariable analysis	
			RR (95% CI)	*p*	RR (95% CI)	*p*
Age	<=60	89	0.828 (0.456–1.506)	0.537	0.800 (0.424–1.510)	0.492
	>60	30				
Tumor size (cm)	<5	62	1.976 (1.183–3.301)	0.009	1.153 (0.628–2.116)	0.646
	> = 5	57				
Serum AFP, ng/ml	<=20	46	1.876 (1.080–3.258)	0.026	1.853 (1.002–3.427)	0.049
	>20	73				
Tumor number	1	97	2.840 (1.605–5.025)	<0.001	2.409 (1.292–4.491)	0.006
	>1	22				
Tumor encapsulation	No	58	0.594 (0.357–0.987)	0.044	0.593 (0.342–1.029)	0.063
	Yes	61				
Microvascular invasion	No	88	3.585 (2.140–6.006	<0.001	1.928 (1.100–3.379)	0.022
	Yes	31				
Lymph node metastasis	No	114	10.727 (3.909–29.439)	<0.001	5.862 (1.854–18.538)	0.003
	Yes	5				
Edmondson-Steiner grade	ES = 1 ~ 2	88	1.366 (0.786–2.373)	0.269	1.068 (0.569–2.004)	0.838
	ES = 3 ~ 4	31				
NAT 10 expression (weak v.s. strong)	0–1	18	6.203 (1.922–20.017)	0.002	5.201 (1.492–18.138)	0.010
	2–3	101				

NAT10 expression was determined by immunohistochemical staining as described in the Methods. Clinico-pathological factors were recorded as mentioned above, and the overall survival of patients was acquired by postoperative follow-up. The univariate analysis suggested that tumor size, tumor number, vascular invasion, lymph node metastasis, and NAT10 expression levels were associated with the overall survival of HCC patients. Then, we employed multivariate Cox regression analysis to identify factors that were independently correlated with patient survival. Tumor size was eliminated, and the remaining factors, including vascular invasion, tumor numbers, lymph node metastasis, and strong expression of NAT10, were identified as independent prognosis risk factors

### Increased NAT10 expression level is correlated with p53 protein level in HCC

**Fig. 3:**
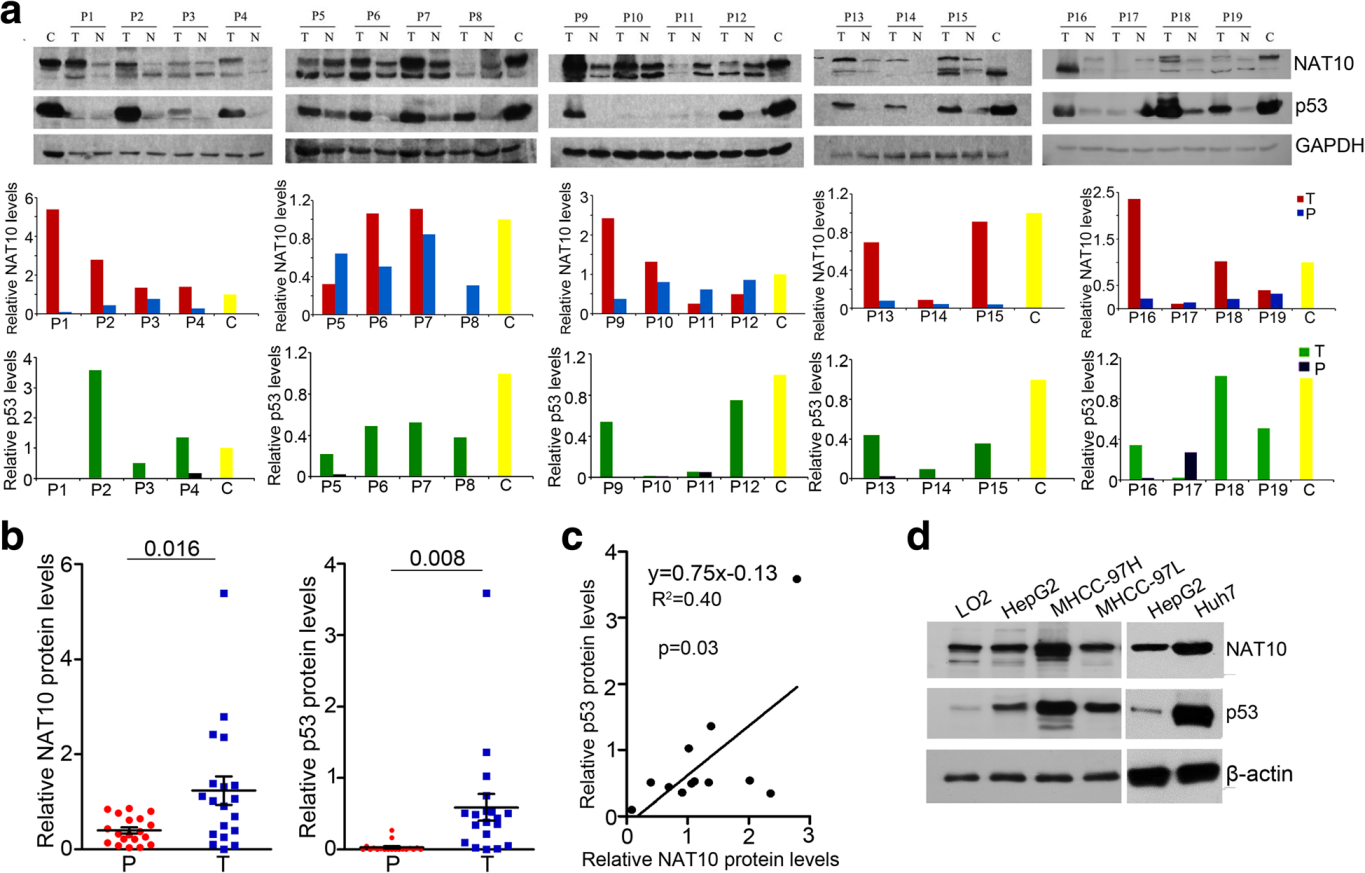
Expression of NAT10 increases in parallel with p53 in human HCC tissues. **a** NAT10 was upregulated in HCC tissues. Proteins extracted from 19 pairs of freshly frozen HCC tissues and paired adjacent non-cancerous tissues were subjected to western blotting with anti-NAT10 and anti-p53 antibodies. GAPDH was used as a loading control (T, cancer tissue; P, pericancerous tissue). **b** Summary of NAT10 and p53 expression in human HCC tissues and noncancerous tissues (T, cancer tissue; P, pericancerous tissue). **c** The positive correlation between the amounts of p53 protein and of NAT10 protein was tested with a Pearson correlation test. **d** NAT10 and p53 expression in HCC cell lines. Cell extracts were prepared from different human HCC cell lines as indicated. Proteins from the extracts were subjected to western blotting for the evaluation of NAT10 and p53 levels. Beta-actin was evaluated as a loading control

A previous study had demonstrated that NAT10 regulates p53 activation [26] and that p53 is frequently mutated in HCC [14]. Therefore, we next investigated whether NAT10 regulates mutant p53 activity in HCC. We compared the NAT10 and p53 protein levels from surgically removed human HCC samples by using immunoblotting. As shown in Fig. 3a and b, p53 was upregulated in 16 of 19 (84.2%) tumor samples, indicating that these tumor samples carry p53 mutations. Notably, we found that NAT10 and p53 levels was positively correlated (r^2^ = 0.4, *p* = 0.03) in the tumor samples with co-upregulation of NAT10 and p53 (Fig. 3c). NAT10 protein levels were also positively correlated with p53 in the HCC cell lines (Fig. 3d). Together, the results above indicate that increased NAT10 expression is correlated with p53 level in HCC.

### NAT10 enhances mutant p53 stability

**Fig. 4:**
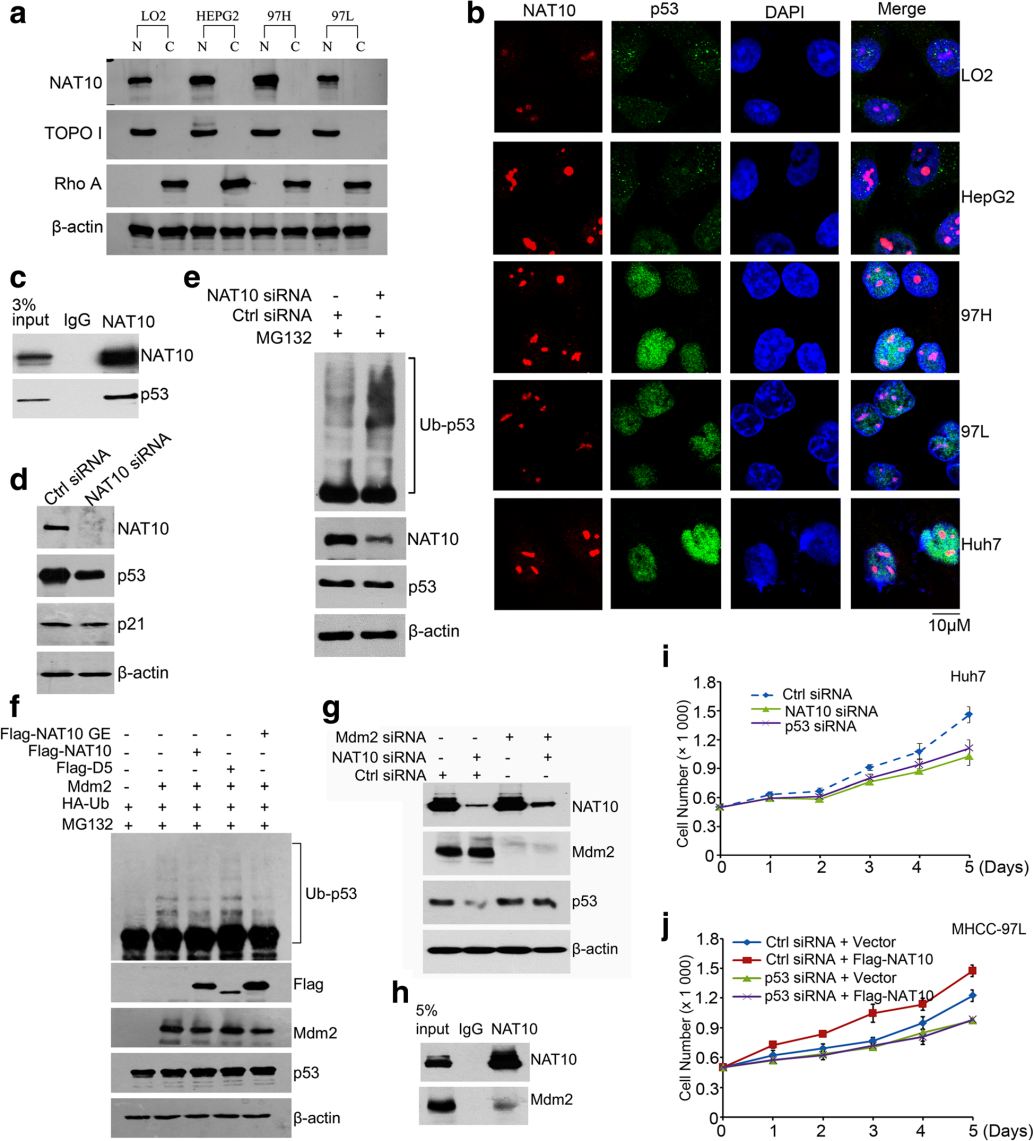
NAT10 stabilizes mutant p53 by counteracting Mdm2 action. **a** LO2, HepG2, MHCC-97H and MHCC-97 L cells were harvested and fractionated. Fractions were then immunoblotted with the indicated antibodies. (C, cytoplasmic; N, nuclear) **b** HCC cells were seeded on coverslips and stained with anti-NAT10 and anti-p53 antibodies. Nuclei were stained with DAPI. Fluorescence images were photographed under confocal microscopy. **c** Huh7 cell lysates were immunoprecipitated with control IgG or anti-NAT10 antibodies. The immunoprecipitates were subsequently immunoblotted with the indicated antibodies. **d** Huh7 cells were transfected with the indicated siRNAs. Seventy-two hours later, the total proteins were analyzed by western blotting for the indicated proteins. **e** Huh7 cells were transfected with the indicated siRNAs and treated with MG132 for 4 h before harvest. The whole cell lysates were analyzed by western blotting for the indicated antibodies. **f** Huh7 cells were transfected with the indicated plasmids. Forty-eight hours later, cells were harvested after MG132 treatment, and the whole cell lysates were analyzed by western blotting for the indicated antibodies. (NAT10 GE: NAT10 mutant lacking acetyltransferase activity; NAT10 D5: NAT10 mutant lacking ubiquitin ligase activity) **g** Huh7 cells were transfected with the indicated plasmids. Forty-eight hours later, cells were harvested and lysed. The whole cell lysates were analyzed by western blotting for the indicated antibodies. **h** Huh7 cell lysates were immunoprecipitated with control IgG or anti-NAT10 antibodies. The immunoprecipitates were subsequently immunoblotted with the indicated antibodies. **i** Huh7 cells transfected with the indicated siRNAs were plated in 96-well plates, and cell proliferation was then quantified at the indicated time points. **j** MHCC-97 L cells transfected with the indicated siRNAs or vectors were plated in 96-well plates, and cell proliferation was then quantified at the indicated time points as described in Methods

To understand the molecular mechanism by which NAT10 regulates mutant p53 in HCC, we investigated whether NAT10 interacts with mutant p53. Extract from cytoplasm and nucleus were fractionated and subjected to Western blotting to evaluate NAT10 expression. As shown in Fig. 4a, NAT10 was detected in the nuclear extracts. Immunofluorescence staining showed that NAT10 was partially colocalized with p53 in the nucleoli (Fig. 4b). Co-immunoprecipitation confirmed that NAT10 bound to mutant p53 in the HCC cell line Huh7 carrying the p53 mutation (Fig. 4c). These findings indicate that NAT10 interacts with mutant p53.

Given the fact that NAT10 regulates p53 activity and in light of our findings that NAT10 level is correlated with mutant p53 level in HCC, we hypothesized that NAT10 also regulates mutant p53 stability. In agreement with our hypothesis, depletion of NAT10 decreased mutant p53 levels; however, no alteration of p21 levels was observed (Fig. 4d). This result was consistent with previous reports that mutant p53 loses the ability to activate wild-type p53 target genes [28, 29]. Furthermore, knockdown of NAT10 increased ubiquitination of mutant p53 (Fig. 4e), indicating that NAT10 regulates mutant p53 ubiquitination and stability. Recent reports suggest that mutant p53 is still under the regulation of Mdm2 [30, 31] and NAT10 modulates Mdm2 activity. Thus, we next analyzed whether NAT10 regulates p53 stability by counteracting Mdm2 action. As shown in Fig. 4f, ectopic expressed Mdm2 could enhance mutant p53 ubiquitination, indicating that Mdm2 could still target mutant p53 to decompose. Importantly, coexpression of NAT10 counteracted the Mdm2-induced ubiquitination of mutant p53 (Fig. 4f). Moreover, the deletion mutant NAT10-D5, which lost the ability to inhibits Mdm2 activity, failed to do so (Fig. 4f, lane 3 vs. lane 4). In addition, NAT10 had no effect on mutant p53 stability in Mdm2-depleted cells (Fig. 4g). The interaction between Mdm2 and NAT10 was further verified by co-immunoprecipitation (Fig. 4h). Taken together, these data indicated that NAT10 regulates mutant p53 stability through counteracting Mdm2 action. Given that mutant p53 often displays acquisition of the ability to potentiates cell proliferation [32], we investigated whether increased NAT10 in cells with mutant p53 could be advantageous to cell proliferation, in contrast to cells with wild-type p53. As expected, downregulation of NAT10 in Huh7 cells resulted in decreased cell proliferation (Fig. 4i). The results were confirmed in MHCC-97 L cells which also carry mutant p53. Overexpression of NAT10 in MHCC-97 L cells enhanced cell proliferation, while ectopic expression of NAT10 had little effect on cell proliferation in p53-depleted MHCC-97 L cells (Fig. 4j). Thus, NAT10 promotes cell proliferation in cells expressing mutant p53. Together, our findings demonstrated that NAT10 regulates mutant p53 and promotes cell proliferation in cells carrying mutant p53.

## Discussion

NAT10 has been observed to function in a variety of cellular processes which are vital to cell growth and proliferation [33, 34]. Besides, it has been indicated that NAT10 involves in affecting the nuclear architecture for the recent work found that NAT10 is the target of the small molecule “Remodelin” which can be used to treat premature aging syndromes by correcting nuclear architecture [25]. Moreover, NAT10 is downregulated in human colorectal cancer [26]. Thus, functional studies of NAT10 will be helpful for the further study of the development and occurrence of cancer. The present study provides experimental evidences that NAT10 is overexpressed in HCC and that NAT10 level is positively correlated with tumor stage. Significantly, shortened OS was observed in correlation with strong NAT10 expression. Multivariate Cox regression analysis further confirmed that NAT10 expression is an independent prognostic factor of HCC. The results from our study cohort suggest that NAT10 may be a tumor promotive factor in HCC occurrence and development. Therefore, we propose that protein quantification of NAT10 in HCC by immunoblotting or immunostaining could be used in combination with pathological examination to predict the biological behaviors of HCC. The combination might be useful in the optimization of personalized treatment.

Previously, we have demonstrated that NAT10 is downregulated in colorectal cancer samples and that NAT10 inhibits cell proliferation and colony formation in cells expressing wild-type p53, indicating that NAT10 could inhibit tumorigenesis through regulating p53 [26]. Here, we report that NAT10 also promotes cell proliferation in cells expressing mutant p53 and increased NAT10 expression correlates with p53 levels in HCC. It is noteworthy that most tumors were observed overexpression of mutant p53, including HCCs [9]. Nevertheless, the underlying mechanisms remain unclear. Recent evidences indicated that the downregulated Mdm2 may be one of the causes for overexpression of mutant p53 [30]. Our results suggest that NAT10 increases mutant p53 stability and promotes cell growth in Huh-7 cells carrying mutant p53. Further, we observed that NAT10 inhibits Mdm2-mediated p53 ubiquitination in Huh-7 cells, indicating that NAT10 regulates both wild-type p53 and mutant p53 stability through counteracting Mdm2 actin. Besides, another chaperone-associated E3 ligase, CHIP was believed to play a vital role for mutant p53 degradation [35]. Thus, it’s unknown whether NAT10 regulates CHIP activity in mutant p53 degradation.

Depletion of NAT10 promotes cell proliferation in cells with wild-type p53 background but decreases cell growth in Huh-7 cells carrying mutant p53. Importantly, NAT10 is overexpressed in HCCs and overexpression of NAT10 is correlated with shortened survival. Moreover, previous study reported that NAT10 plays an important role in the growth of a subtype of epithelial ovarian cancer with poor prognosis [36]. Therefore, the role of NAT10 in tumorigenesis and cancer progression may vary in different types of tumors. Further investigations, especially animal experiments, are highly necessary to understand the pathophysiological role of NAT10 in tumor initiation and progression.

Most tumors especially HCC have observable genetic changes. Mutated or functional deficient p53 was one of the most prevalent events observed and mutations of p53 are mainly missense point mutations in the DNA-binding domain [37, 38]. Such mutations abrogate transcription of p53 target genes, thereby disrupting the tumor-suppressing activities of p53. Additionally, the proteins generated by mutated *Tp53* gene acquire oncogenic functions by endowing cells with proliferation and growth advantage [39]. In vivo experiments have revealed that tumors in mice with mutant p53 had the characteristic of higher malignancy, rapid development and more invasive compared with wild type or null p53 mice [40, 41]. Thus, novel strategies are being developed aiming at tumors with mutant p53 [17, 42]. Abrogation of histone deacetylase HDAC6-binding could cause the heat-shock proteins disassociated from mutant p53, with the result that mutant p53 is easier to be degraded by Mdm2. Thus, HDAC inhibitors such as SAHA has the potential in promoting mutant p53 degradation and removing mutant p53 [43, 44]. Small molecule activators of Sirt1 have also been used to reduce mutant p53 levels [45]. NAT10 is upregulated in HCC, and it enhances p53 stability, indicating that NAT10 might be a potential target in HCC therapy. Abrogation of the interaction between NAT10 and p53 would be beneficial for tumor therapy of hepatic cancers carrying p53 mutations. These possibilities need to be examined in future studies.

## Conclusions

Our study demonstrated that NAT10 is upregulated in HCC and that increased NAT10 expression levels are associated with shortened patient survival. Moreover, NAT10 interacts with mutant p53 and increases its stability, resulting in increased cell proliferation in HCC cells. These results indicate that NAT10 is a potential therapeutic candidate for p53-mutated HCC.

## Abbreviations

CHIP: Carboxyl terminus of Hsc70-interacting protein
HADC: Histone deacetylase
HCC: Hepatocellular carcinoma
Hsp90: Heat shock protein 90
IHC: Immunohistochemistry
Mdm2: Murine double minute2
NAT10: N-acetyltransferase 10
OS: Overall survival
PBS: Phosphate buffered solution
SAHA: Suberoylanilide hydroxamic acid
